# Effects of the Operating Ambiance and Active Layer Treatments on the Performance of Magnesium Fluoride Based Bipolar RRAM

**DOI:** 10.3390/nano12040605

**Published:** 2022-02-11

**Authors:** Nayan C. Das, Minjae Kim, Dong-uk Kwak, Jarnardhanan R. Rani, Sung-Min Hong, Jae-Hyung Jang

**Affiliations:** 1 School of Electrical Engineering and Computer Science, Gwangju Institute of Science and Technology, Gwangju 61005, Korea; nayan@gist.ac.kr (N.C.D.); min7kim9@gist.ac.kr (M.K.); kwakdd09@gm.gist.ac.kr (D.-u.K.); rani@gist.ac.kr (J.R.R.); smhong@gist.ac.kr (S.-M.H.); 2 School of Energy Engineering, Korea Institute of Energy Technology, Naju 58330, Korea

**Keywords:** bipolar, operating environment, annealing, filament type resistive switching, RRAM

## Abstract

This study investigates switching characteristics of the magnesium fluoride (MgF_x_)-based bipolar resistive random-access memory (RRAM) devices at different operating ambiances (open-air and vacuum). Operating ambiances alter the elemental composition of the amorphous MgF_x_ active layer and Ti/MgF_x_ interface region, which affects the overall device performance. The experimental results indicate that filament type resistive switching takes place at the interface of Ti/MgF_x_ and trap-controlled space charge limited conduction (SCLC) mechanisms is dominant in both the low and high resistance states in the bulk MgF_x_ layer. RRAM device performances at different operating ambiances are also altered by MgF_x_ active layer treatments (air exposure and annealing). Devices show the better uniformity, stability, and a higher on/off current ratio in vacuum compared to an open-air environment. The Ti/MgF_x_/Pt memory devices have great potential for future vacuum electronic applications.

## 1. Introduction

Resistive switching random access memory (RRAM) devices are one of the emerging non-volatile memory (NVM) technologies with two terminal metal/insulator/metal (MIM) structures [1,2]. The simple MIM structures make RRAMs integrated into dense crossbar arrays and traditional, complementary metal-oxide-semiconductors (CMOS) [2]. RRAM stores data by using different resistance states. Binary information (“0” and “1”) can be stored within one device cell using high and low resistance states, respectively. More information can be stored within a single device cell using multiple resistance states for multi-level information storage. For example, four different pieces of information (“00”, “01”, “10”, and “11”) can be stored within one device cell using four different resistance states [3]. Besides data storage, RRAM devices show remarkable similarities to biological synapses, dendrites, and neurons at both the physical mechanism level and unit functionality level. These similarities make the RRAM-based neuromorphic computing a promising technology for future artificial intelligence [4]. Additionally, RRAMs show potential for next-generation high-density NVMs, cryogenic computing, and artificial neural computing due to their high programming speed, low-voltage operation, high scalability and simple fabrication/integration processes [1,2,3,4,5]. Even though significant performance improvements in the RRAM device have been achieved, one remaining drawback is the large parameter variability, whose cause has been ascribed to moisture present in the atmospheric environment [6].

Studies show that the absorption of moisture or any gas usually happens by direct contact of the device with the environment, sidewall diffusion, and encapsulating layers. It can also happen during the device fabrication process [6,7]. The moisture absorption from the ambient air in oxide materials changes the characteristics of the metal-oxide-semiconductor device, leading to the changes in their electrochemical and resistive switching performance [6,8,9,10,11]. Moreover, as the device size becomes smaller, the effects of gaseous ambiance become stronger due to the large specific surface area. Thus, understanding the effect of the surrounding conditions on the RRAM device performance is strongly required to design and control the features of nanoscale RRAM devices [1].

Several studies investigated the effects of surroundings by varying air, oxygen, and nitrogen partial pressure on RRAM devices’ performance [1,12,13]. Some studies demonstrated that it is not possible to achieve electroforming in a vacuum for SiO_2_, Ta_2_O_5_, and HfO_2_-based devices [6,7,11,14]. Other studies require an electroforming process in open-air to activate the resistive switching properties before measuring device performance in vacuum [15,16,17,18,19,20,21]. Nevertheless, most studies failed to report the device’s cycling property and stability in a vacuum. These limitations, as mentioned above, need to be overcome to develop a reliable RRAM device that works in a vacuum and in the open air. Thus, it is necessary to comprehensively study the interaction of the RRAM device in open-air and vacuum settings.

To date, very few oxygen vacancy-based materials have been investigated as an active layer of RRAM devices in a vacuum. The operating ambiance heavily influences the oxygen vacancy-based active layer and the overall performance of the memory devices due to a large amount of oxygen available in the atmosphere. Exploring the alternative anion vacancy-based material, which is less influenced by the operating ambiance, can be one of the ways to overcome the limitations of oxygen vacancy-based RRAMs operating in a vacuum.

Biodegradable magnesium fluoride (MgF_x_) has been utilized in many eco-friendly electronics [22]. MgF_x_, being a wide bandgap (11.3 eV) insulator, has also shown potential for RRAM devices [22,23]. Our recent work on Ti/MgF_x_/Pt devices demonstrated electroforming-free bipolar resistive switching behaviour in the open-air environment [5]. The influence of operating ambiance on fluoride vacancy-based RRAM is yet to be explored. Thus, it is crucial to investigate the vacuum performance of the MgF_x_-based RRAM.

This work reports the fluoride vacancy-based Ti/MgF_x_/Pt RRAM devices operable in a vacuum without pre-treatment in the open-air environment. The influence of the operating environments was observed on the device performances. Nevertheless, the device showed stable and more uniform performance in a vacuum than in an open-air environment. The electroforming process is dependent on the operating ambiance. Structural, elemental, and compositional characteristics of the MgF_x_ thin film are systematically investigated in different environments to optimize the RRAM device performances at different operating ambiances. MgF_x_ active layer treatments, such as annealing and air exposer, worsen the device performance in the open-air but improve the device performance in a vacuum.

This study revealed that the performance of Ti/MgF_x_/Pt RRAM devices varies at different operating environments due to the variation in elemental compositions of the Ti/MgF_x_ interface region. Finally, mechanisms are presented in detail with proper conduction and resistive switching model.

## 2. Materials and Methods

To fabricate the Ti/MgF_x_/Pt devices, a 150-nm-thick Pt bottom electrode with a thin adhesion layer of Ti is deposited on SiO_2_/Si substrate by electron beam (e-beam) evaporation. The 50-μm-radius circular shadow mask was utilized to pattern 50-nm-thick MgF_x_ and 150-nm-thick Ti during deposition without exposing the active layer to the open-air environment. MgF_x_ thin films were deposited by e-beam evaporation under a base pressure of 1 × 10^−6^ Torr on various substrates. The evaporation rate was maintained constant at 2 Å/s. The substrate temperature was ambient.

To investigate the effect of active layer treatments, Ti/MgF_x_/Pt devices were also fabricated with a conditioned (5 min annealing on hot plate at 50 °C, 1-h ambient exposure) MgF_x_ layer. For scanning electron microscope (SEM) analysis, X-ray diffraction (XRD) analysis, X-ray photoelectron spectroscopy (XPS), and Fourier transform infrared (FTIR) absorbance spectroscopy measurement, 50-nm and 1-μm-thick MgF_x_ films were grown separately.

In an open-air laboratory environment, the electrical characteristics of the memory devices were measured using a semiconductor parameter analyzer (HP-4155A, Palo Alto, CA, USA). Voltage was applied directly to the top electrode, while the bottom electrode was grounded. The electrical characteristics of RRAM devices in a vacuum environment were measured using MS-TECH Vacuum Chamber Probe Station (<10^−3^ torr) (Hwaseong-si, Gyeonggi-do, Korea).

The device fabrication process is straightforward. A minimum three different batches of samples (a batch consisting of more than twenty devices) for each sample type were analyzed to confirm the reproducibility. More than fifty devices were measured at each condition to confirm the observations and conclusions. The range of device-to-device variation was smaller than cycle-to-cycle variations because there were few process variables involved in the device fabrications and each process condition was well controlled.

## 3. Results and Discussion

### 3.1. MgF_x_ Film Characteriazations

**Figure 1:**
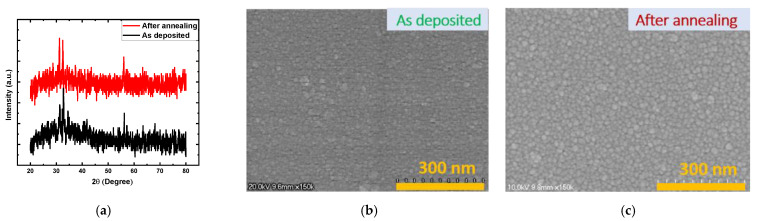

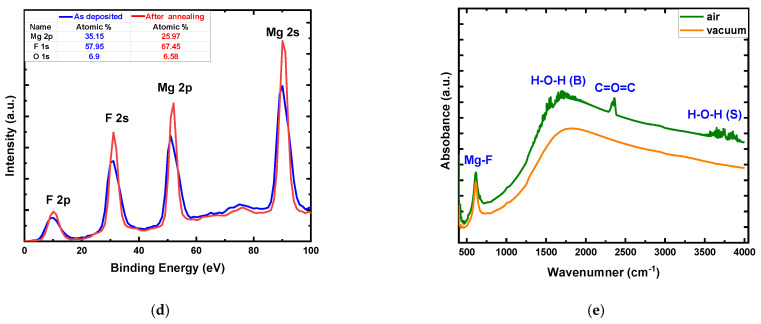
Structural and compositional analysis of MgF_x_ thin film: (**a**) XRD pattern of the as−deposited and annealed MgF_x_ films; SEM surface images of the (**b**) as−deposited and (**c**) annealed films; (**d**) XPS analysis with atomic percentages of the as-deposited and post-deposition annealed MgF_x_ films; (**e**) FTIR absorbance spectra measured in open air and vacuum environment.

Ti/MgF_x_/Pt device performances are mainly governed by the properties of the active MgF_x_ layer. To understand the operating mechanisms of the devices, it is necessary to analyze the MgF_x_ layer thoroughly. Figure 1 shows XRD pattern, SEM image, XPS analysis, and FTIR absorbance spectroscopy measurement results for the MgF_x_ thin films.

Figure 1a shows the XRD patterns of the as-deposited and post-deposition annealed MgF_x_ thin films. The XRD analysis shows that the films were amorphous. The SEM images of these MgF_x_ thin films are shown in Figure 1b,c. As-deposited MgF_x_ thin film shows the formation of small grains. Post-deposition annealed MgF_x_ thin film also shows the formation of grains comparatively bigger than those of the as-deposited film. The crystal structure of the MgF_x_ thin films can be manipulated from amorphous to crystalline by applying substrate temperature from ambient to 300 °C [24,25]. The XRD and SEM analysis reveal that the defect-rich amorphous granular structured MgF_x_ layer was successfully fabricated by keeping the substrate temperature at ambient temperature during e-beam deposition.

Figure 1d shows XPS analysis with characteristic peaks and atomic percentages of the as-deposited and post-deposition annealed MgF_x_ films. Both films show the presence of Mg and F by the characteristic Mg 2p peak with a slight change in positions at around 52 eV. The shift in peak positions indicates the difference in the compositions. A small amount of oxygen is also found [6,22]. However, there was a significant change in the atomic ratio of Mg to F between the as-deposited and post-deposition annealed MgF_x_ films found by curve-fitting and area analysis. The Mg/F ratio in the as-deposited MgF_x_ film was around 1:1.65, which shows the existence of fluoride vacancies in the film. In contrast, the Mg/F ratio of the post-deposition annealed MgF_x_ film was approximately 1:2.60. The increase of fluoride implies that F atoms were not necessarily missing but dislocated in interstitial sites and at grain boundaries of the amorphous film during deposition [26,27]. During annealing, recombination occurs and dislocated F atoms come back to their proper positions, which results from interactions between an electron, an F vacancy, and a dislocated fluorine atom [26,27]. Thus, grain size increases.

Figure 1e shows MgF_x_ thin-film FTIR absorbance spectra measured in the open air and vacuum environment. The stable Mg–F bond characteristic absorbance peak was found at 613 cm^−1^ for both the measurement conditions. However, many weak absorption peaks were observed between 3800~3500 cm^−1^ and 1700~1450 cm^−1^ in open-air measurement conditions due to the weak binding between Mg^2+^ sites and different vibrational modes (stretching and bending) of hydroxyl groups [28,29,30]. These hydroxyl groups indicate that H_2_O is absorbed at the surface of the amorphous MgF_x_ during fabrication [6,26,30]. At around 2375~2385 cm^−1^, a weak CO_2_ vibration band was also detected [30]. All the hydroxyl groups and CO_2_ are easily removed from the amorphous MgF_x_ thin film in the vacuum condition [29,31,32]. The availabilities of these weakly bonded groups heavily affect the surface chemistry of the amorphous MgF_x_ active layer, as well as the Ti/MgF_x_ interface [6,7,33,34].

### 3.2. Electrical Characteristics of Ti/MgF_x_/Pt Device

**Figure 2:**
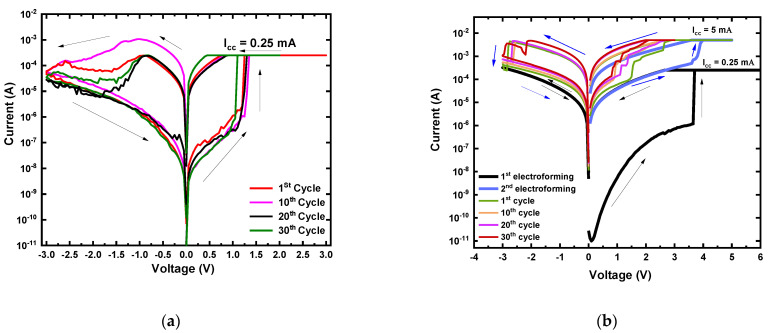
Typical I−V characteristics of as-deposited MgF_x_ based Ti/MgF_x_/Pt memory devices. (**a**) in open−air; (**b**) in vacuum.

In an open-air environment, the current-voltage (I–V) measurement was carried out for the devices with a 25 μm radius by applying double sweep DC voltage in the sequence of 0 V → +3 V → 0 V → −3 V → 0 V under the compliance current (I_cc_) of 0.25 mA. Figure 2a shows I–V characteristics of an as-deposited MgF_x_-based Ti/MgF_x_/Pt memory device in an open-air environment. Electroforming free bipolar resistive switching behavior of the Ti/MgF_x_/Pt devices was observed with an on/off ratio >10^2^. The electroforming-free characteristics is caused by the combined effects of sufficient internal fluoride vacancies and the presence of a small amount of O–H groups at the surface of the amorphous MgF_x_ active layer [5]. The O–H groups provide additional charges and facilitate the formation of anion vacancies at the interface of the Ti/MgF_x_ [6,7,33,34]. A detailed study of the electroforming-free bipolar resistive switching behavior of the Ti/MgF_x_/Pt devices is reported separately [5]. The area-independent (device radii of 25, 50, 150, and 225 μm) voltages (V_SET_ and V_REST_) and currents (I_LRS_ and I_HRS_) imply that filament type resistive switching takes place in Ti/MgF_x_/Pt devices [5]. The thickness independence of V_SET_ and V_REST_ implies that the resistive switching mainly occurred at the top electrode/dielectric interface [5].

#### 3.2.1. Effects of Operating Environment on Device Performance

I–V measurement of the devices was carried out in a vacuum (<10^−3^ torr) to investigate the effects of the operating environment on the as-deposited MgF_x_ based Ti/MgF_x_/Pt device performance. Figure 2b shows I–V characteristics of a Ti/MgF_x_/Pt memory device in a vacuum environment.

The first double sweep DC voltage was applied in the sequence of 0 V → +5 V → 0 V → −3 V → 0 V. The pristine device was in the high resistance state (HRS) vacuum condition.

When a positive bias voltage was applied, the current increased gradually as the voltage increased, and jumped sharply up to the I_cc_ at electroforming voltage (V_Forming_) around +4 V. When a negative bias voltage was applied, the device maintained in low resistance state (LRS). The I–V curve did not show any resistive switching characteristics (thick black color with black arrows in Figure 2b). However, by increasing the I_cc_ to 5 mA, the device showed bipolar resistive switching properties. Electroforming completed around +4 V during positive bias voltage, and the device reached new LRS (thick blue color with blue arrows in Figure 2b). After applying the negative bias voltage, the resistance state of the device came back to the new HRS. From the third cycle, the sequence was 0 V → +3 V → 0 V → −3 V → 0 V. After electroforming, the SET process occurred at around +1 V, which was SET voltage (V_SET_) lower than the V_Forming_. Similarly, the RESET process occurred at RESET voltage (V_RESET_) of approximately −2.5 V. The I_LRS_ and I_HRS_ values of the device were read at +0.50 V (V_Read_).

In a vacuum environment, the pristine Ti/MgF_x_/Pt device needs an electroforming process to activate resistive switching properties and exhibiting higher initial resistance (~10 GΩ) than the initial resistance (~10 MΩ) in an atmospheric environment where the device was electroforming-free. Overall device performances changes in a vacuum with the reference of an atmospheric environment as follow: (1) Fluctuation of I–V curves decreases, (2) SET voltage slightly decreases from +1.25 V to +1.0 V, (3) RESET voltage increases from 0.9 V to 2.5 V, (4) SET and RESET current increases, (5) On/off ratio decreases from over ~10^3^ to over 10.

In the vacuum environment, removing weakly bonded hydroxyl groups and CO_2_ from the surface of the amorphous MgF_x_ thin film made the active layer more resistive, and the overall initial resistance of the Ti/MgF_x_/Pt device increased by a factor of 3. Thus, the defect-rich amorphous MgF_x_ layer with fluoride vacancies was insufficient to activate the resistive switching properties in the device without electroforming. The electroforming process was applied with the I_cc_ of 0.25 mA. However, resistive switching properties were not activated, which can be attributed to the incomplete initial formation of the conduction filament (CF).

After increasing the I_cc_ to 5 mA, the resistive switching properties of the devices were activated. With the increase of I_cc_, the size of the CF increased, and the resistance of the CF decreased. As a result, the I_LRS_ increased. It was also found that I_HRS_ and V_RESET_ values have increased with the higher I_cc_ due to the higher power requirement to dissolve the CF with a larger diameter [35,36,37].

After electroforming, the CF dissolved partially during the RESET process, and the device reached new HRS, which is less resistive than the initial HRS. Further SET/RESET processes happen in the weakest point of the CF by partial reconstruction/rupture, which needed smaller V_SET_ in a vacuum compared to the atmospheric environment [35].

#### 3.2.2. Conduction and Resistive Switching Mechanism in Vacuum Environment

##### Log(I)−Log(V) Curves Analysis

**Figure 3:**
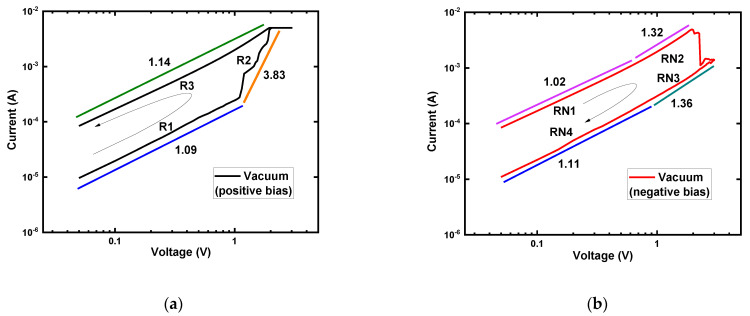
Log (I) − log (V) characteristics of Ti/MgF_x_/Pt memory devices with I_cc_ = 5 mA in vacuum environment. (**a**) Positive bias voltage region; (**b**) Negative bias voltage region with slopes of different parts.

After electroforming, a typical I–V curve was replotted as log(I)−log(V) to investigate the conduction mechanism of the Ti/MgF_x_/Pt device in the vacuum environment. The curve fittings results are shown in Figure 3. The positive voltage regions in HRS and LRS were divided into R1, R2, and R3, as shown in Figure 3a. The negative bias voltage regions were also divided into RN1, RN2, RN3, and RN4, as shown in Figure 3b.

In the positive voltage region until the SET voltage, the slopes of the fitting lines for both HRS (R1: 1.09) and LRS (R3: 1.14) were close to 1, which indicates that ohmic conduction (I∝V) dominated in these regions. As the voltage increased from +1 V to +2 V, the slopes of HRS (R2: 3.83) gradually increased. At higher voltages, the conduction mechanism followed the Child’s law (I~V^n^). The I–V characteristics in the negative voltage region also showed a similar pattern, but with sharp changes from HRS to LRS (Figure 3b). These conduction characteristics of LRS and HRS indicate the trap-controlled space charge limited conduction (SCLC) mechanism, and the resistive switching was caused by controllable transformation from charge trapping/de-trapping to filamentary conduction [38,39,40,41,42].

#### 3.2.3. Effects of Active Layer Treatments on Device Performance in Different Environment

The performance of the RRAM device can be regulated by modifying active layer properties. In this work, MgF_x_ layer properties were modified by air exposure and annealing. The modified MgF_x_ layer properties and their overall effects on device performance were investigated in different operating environments.

##### Effects of Air Exposed MgF_x_ Active Layer

**Figure 4:**
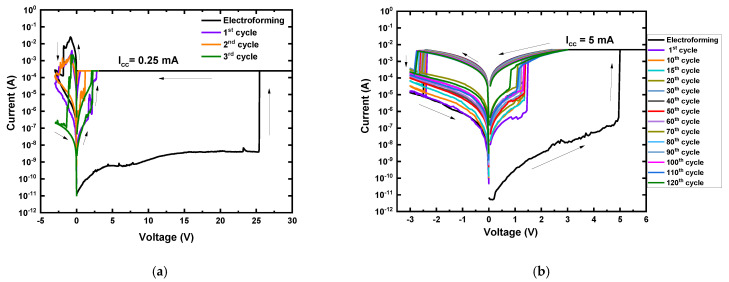
Typical I−V characteristics of post-deposition air−exposed MgF_x_ based Ti/MgF_x_/Pt memory devices. (**a**) in open−air; (**b**) in vacuum.

The MgF_x_ active layer was intentionally exposed to the open-air for one hour in the laboratory room environment to explore the effect of air exposure. After device fabrication, performance was measured in open-air and vacuum environments, shown in Figure 4.

In an open-air environment, the I–V measurement of the air-exposed MgF_x_ thin-film-based memory device is shown in Figure 4a. The air-exposed device exhibited three orders of magnitude higher initial resistance (~GΩ) than the as-deposited device (~MΩ) in an open-air environment. The device showed an electroforming characteristic at around 25 V, when a double sweep DC voltage was applied in the sequence of 0 V → +30 V → 0 V → −4 V → 0 V and I_cc_ of 0.25 mA. When a negative voltage was applied, the RESET process occurred at around −2.5 V. The sequence was 0 V → +3 V → 0 V → −3 V → 0 V after electroforming. The device showed resistive switching properties, but with fluctuations.

However, the air-exposed MgF_x_ thin-film-based Ti/MgF_x_/Pt memory devices showed reversible resistive switching properties with improved stability and on/off current ratio >10^2^ in a vacuum environment. Figure 4b shows the first sequence 0 V → +25 V → 0 V → −5 V → 0 V for electroforming. After the electroforming process, the sequence was 0 V → +3 V → 0 V → −3 V → 0 V with I_cc_ of 5 mA. The LRS is more stable than HRS. These phenomena can be attributed to the exposure in open-air and the reversible adsorption effect in vacuum environments shown in FTIR analysis in Figure 1e [29,31,32].

Amorphous MgF_x_ films generally absorb more moisture than crystalline films due to the defects present at their grain boundaries [6,28,29,30]. During the air exposure, the amorphous MgF_x_ film absorbed moisture in the defects (F vacancies) of films. Thus, this air exposure decreased the fluorine vacancy and made the MgF_x_ film more resistive [43]. As a result, air-exposed devices require the higher electroforming voltage to make the initial conducting filament. The device exhibited the higher resistance and, even after electroforming, its performance was not stable, due to insufficient fluorine vacancies.

The reduced fluorine vacancy concentration in the air-exposed amorphous MgF_x_ thin films could be recovered by putting it under vacuum, because water adsorption in porous MgF_x_ thin films is a reversible [29], or at least partially reversible [32], process. Consequently, the air-exposed MgF_x_ thin-film-based Ti/MgF_x_/Pt memory devices mostly recover the fluorine vacancies in a vacuum environment by removing moisture. They show initial resistance similar to devices in a vacuum, a slightly higher electroforming voltage (~5 V), and an almost similar SET and RESET voltage.

##### Effects of Annealed MgF_x_ Active Layer

**Figure 5:**
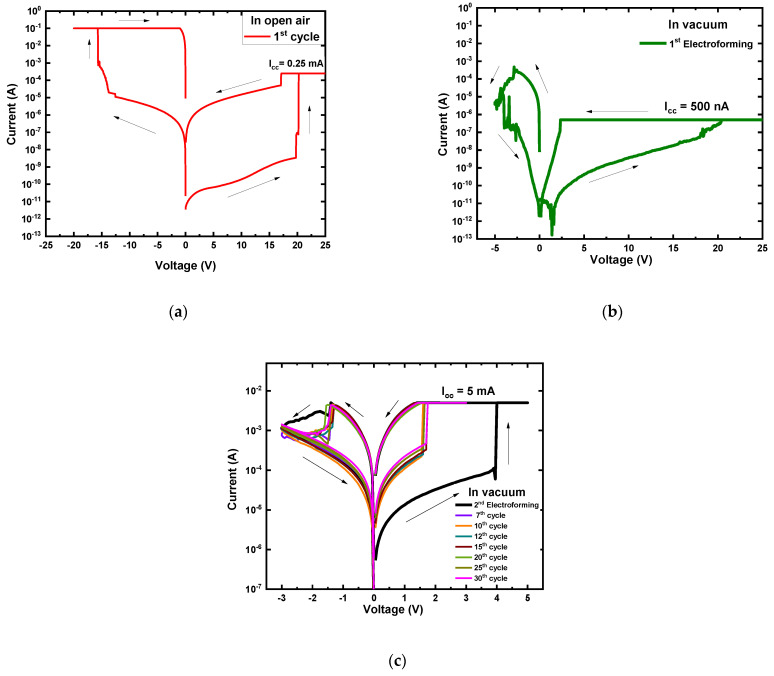
Typical I−V characteristics of post-deposition annealed MgF_x_−based Ti/MgF_x_/Pt memory devices. (**a**) in open−air; (**b**) in vacuum first electroforming; (**c**) in vacuum second electroforming with I_cc_ = 5 mA.

The temperature treatment plays a vital role in the film properties [44,45]. Thin films annealed after deposition generally exhibit improved durability and stability [46,47]. Post-deposition annealed MgF_x_ thin film was utilized to fabricate Ti/MgF_x_/Pt memory devices, and their characteristics were measured in open-air (Figure 5a) and vacuum environment (Figure 5b,c).

In an open-air environment, the I−V characteristics of the device based on the annealed MgF_x_ are shown in Figure 5a. The annealed device exhibited a three orders of magnitude higher initial resistance (~GΩ) than the as-deposited device (~MΩ) in an open-air environment. Under positive biasing conditions, the device showed resistive switching from HRS to LRS at around ~20 V only one time. The device failed to show the RESET process and broke down when a negative voltage was applied.

However, the device based on the annealed MgF_x_ thin-film became more resistive (~TΩ) in a vacuum environment than in open air and showed stable resistive switching memory properties. Nevertheless, a two-step electroforming process was required to stabilize the resistive switching process. The first electroforming process reduced the initial device resistance by soft breakdown to MΩ (similar range as initial as-deposited device). The second electroforming process completed the formation of well-developed CF with similar V_Forming_ (~4 V) as the as-deposited device in a vacuum. Figure 5b shows the first electroforming process of the device. A double sweep DC voltage was applied in the sequence of 0 V → +25 V → 0 V → −5 V → 0 V. The second electroforming was observed for the devices based on the annealed MgF_x_ thin-film, as shown in Figure 5c. Second electroforming was carried out by the sequence of 0 V → +5 V → 0 V → −3 V → 0 V with I_cc_ of 5 mA. The further cycling experiments were carried out in the sequence of 0 V → +3 V → 0 V → −3 V → 0 V from the third cycle. Devices show very stable symmetric bipolar resistive switching properties with SET and RESET voltages around +1.5 V and −1.5 V, respectively.

Due to the annealing, fluorine vacancy-based MgF_x_ (1:1.65) becomes fluorine-rich (1:2.60) with a relatively bigger grain than as-deposited film, as shown in Figure 1b,c. Furthermore, moisture adsorption in the fluorine-rich film makes it unsuitable for forming CF in an open-air environment [6,28,29,30]. As a result, the annealed MgF_x_ thin-film-based device did not show resistive switching properties in an open-air environment.

In a vacuum, removing moisture with step-by-step soft breaking made it possible to form CF; the device showed resistive switching properties.

### 3.3. Comparison of the Resistive Switching Mechanism of Differently Conditioned MgFx Based RRAM in Vacuum

**Figure 6:**
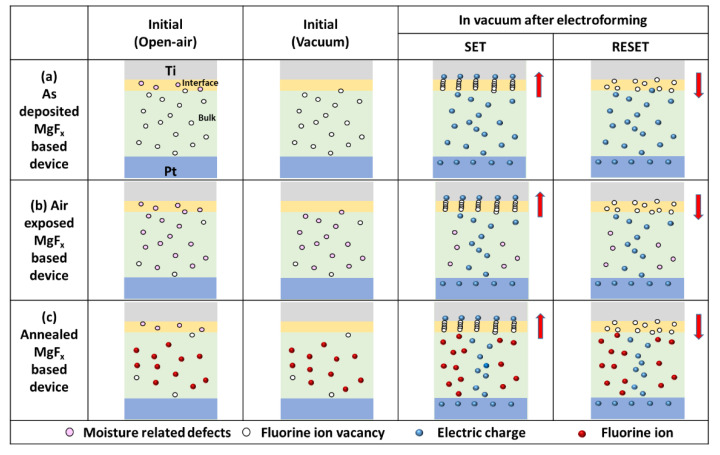
The figure shows the schematic comparison of the resistive switching mechanism in a vacuum; (**a**) as−deposited, (**b**) air−exposed, and (**c**) annealed MgF_x_ based RRAM.

Figure 6 shows the schematic diagram comparing the resistive switching mechanism of as-deposited, air-exposed, and annealed MgF_x_-based RRAM in a vacuum environment.

As-deposited MgF_x_-based RRAM contains a small number of hydroxyl groups (moisture-related defects) at the interface of the Ti/MgF_x_ at the initial stage in open-air. However, those moisture-related defects were removed in a vacuum. The device requires an electroforming process to form a fluorine vacancy-based CF filament at the Ti/MgF_x_ interface of the Ti/MgF_x_/Pt memory device. After electroforming, the RESET and the SET processes are enabled by partial fracture and reconstruction of CF at the interface (Figure 6a). After active layer treatments (air-exposed and annealed), the density of MgF_x_ film increases, which causes significantly increased V_Forming_ [6].

For air-exposed MgF_x_-based RRAM, the MgF_x_ film density increased due to incorporating moisture at the interface and in bulk. However, in a vacuum, most moisture from the interface and some from bulk were removed due to the MgF_x_ reversible adsorption property [29,31,32]. Thus, air-exposed MgF_x_-based RRAM needs lower V_Forming_ in a vacuum than in an open-air environment. At V_Forming_, fluorine vacancy-based CF filament are formed at the interface and pre-existing defects of fluoride vacancies are recovered in the bulk MgF_x_ layer. Once CF is formed by electroforming process, the RESET and the SET process are enabled by partial rupture and restoration of CF at the interface (Figure 6b).

For annealed MgF_x_-based RRAM, the MgF_x_ film density increased due to the recrystallization and rearrangement of chemical composition and becoming a fluorine-rich film. In addition, the incorporated moisture makes the MgF_x_ film more unfavorable for resistive switching in the open air. However, a fluorine vacancy-based conducting path is created in the balk and at the interface by a two-step electroforming process in a vacuum. The resistive switching mechanism, operating by charge trapping and de-trapping in the bulk amorphous MgF_x_ layer and by the formation and rupture of CF at the Ti/MgF_x_ interface region. That is why the device shows more consistent and stable performance (Figure 6c).

## 4. Conclusions

Effects of different operating ambiances (open-air and vacuum) and active layer treatments (air exposer and annealing) on the performance of fluoride vacancy-based Ti/MgF_x_/Pt RRAM devices are investigated. Operating environment and active layer treatments critically regulate the device performance by varying elemental composition of the amorphous MgF_x_ active layer and Ti/MgF_x_ interface region. The presence of hydroxyl groups (moisture) at the interface helps the device perform electroforming-free resistive switching properties in an open-air environment. In a vacuum, the device gets more resistive due to the moisture removal and requires an electroforming process to activate its resistive switching properties.

Overall, device performance in a vacuum is optimized by active layer treatments. The air-exposed MgF_x_-based RRAM shows better stability with an on/off ratio > 10^2^ in a vacuum due to the partial reversible moisture adsorption effect of MgF_x_. The annealed MgF_x_-based RRAM demonstrates symmetric bipolar resistive switching with better uniformity in vacuum due to the combined recrystallization and partial reversible moisture adsorption effect of MgF_x_.
